# An improved workflow for accurate and robust healthcare environmental surveillance using metagenomics

**DOI:** 10.1186/s40168-022-01412-x

**Published:** 2022-12-02

**Authors:** Jiaxian Shen, Alexander G. McFarland, Ryan A. Blaustein, Laura J. Rose, K. Allison Perry-Dow, Anahid A. Moghadam, Mary K. Hayden, Vincent B. Young, Erica M. Hartmann

**Affiliations:** 1 Department of Civil and Environmental Engineering, Northwestern University, Evanston, IL 60208-3109 USA; 2 Department of Nutrition and Food Science, University of Maryland, College Park, USA; 3 Centers for Disease Control and Prevention, Atlanta, USA; 4 Division of Infectious Diseases, Department of Internal Medicine, Rush Medical College, Chicago, USA; 5 Department of Internal Medicine/Division of Infectious Diseases, The University of Michigan Medical School, Ann Arbor, USA

**Keywords:** Environmental surveillance, Metagenomics, Infection prevention, Low biomass, Viability, Quantification, Machine learning, Sequencing depth prediction

## Abstract

**Background:**

Effective surveillance of microbial communities in the healthcare environment is increasingly important in infection prevention. Metagenomics-based techniques are promising due to their untargeted nature but are currently challenged by several limitations: (1) they are not powerful enough to extract valid signals out of the background noise for low-biomass samples, (2) they do not distinguish between viable and nonviable organisms, and (3) they do not reveal the microbial load quantitatively. An additional practical challenge towards a robust pipeline is the inability to efficiently allocate sequencing resources a priori. Assessment of sequencing depth is generally practiced post hoc, if at all, for most microbiome studies, regardless of the sample type. This practice is inefficient at best, and at worst, poor sequencing depth jeopardizes the interpretation of study results. To address these challenges, we present a workflow for metagenomics-based environmental surveillance that is appropriate for low-biomass samples, distinguishes viability, is quantitative, and estimates sequencing resources.

**Results:**

The workflow was developed using a representative microbiome sample, which was created by aggregating 120 surface swabs collected from a medical intensive care unit. Upon evaluating and optimizing techniques as well as developing new modules, we recommend best practices and introduce a well-structured workflow. We recommend adopting liquid-liquid extraction to improve DNA yield and only incorporating whole-cell filtration when the nonbacterial proportion is large. We suggest including propidium monoazide treatment coupled with internal standards and absolute abundance profiling for viability assessment and involving cultivation when demanding comprehensive profiling. We further recommend integrating internal standards for quantification and additionally qPCR when we expect poor taxonomic classification. We also introduce a machine learning-based model to predict required sequencing effort from accessible sample features. The model helps make full use of sequencing resources and achieve desired outcomes.

Video Abstract

**Conclusions:**

This workflow will contribute to more accurate and robust environmental surveillance and infection prevention. Lessons gained from this study will also benefit the continuing development of methods in relevant fields.

**Supplementary Information:**

The online version contains supplementary material available at 10.1186/s40168-022-01412-x.

## Introduction

Effective microbial surveillance in the built environment is increasingly important in infection prevention, given the persistence of pathogens in environmental reservoirs and their potential transmission to patients [1–7] (e.g., carbapenem-resistant *Klebsiella pneumoniae* in sink drains [8, 9]). Furthermore, with the emergent studies showing synergistic relationships among pathogens on hospital surfaces [10] and the possibility for pathogenic bacteria to acquire antibiotic resistance genes from non-pathogenic neighbors [11], it is necessary to expand from targeted surveillance to untargeted methods. Untargeted methods are advantageous in identifying novel or rapidly emerging pathogens [12]. Metagenomics-based techniques are the most promising option to achieve these goals but are currently challenged by several limitations: (1) they are not powerful enough to extract valid signals out of the background noise for low biomass samples, (2) they do not distinguish between viable and nonviable organisms, and (3) they do not reveal the microbial load quantitatively [13, 14].

For challenge 1, adoption of appropriate negative controls has been emphasized [15, 16], along with various bioinformatic filtering tools to remove putative contaminants [17, 18]. While current efforts have largely focused on contamination prevention, increasing the biomass itself remains understudied [12]. Having adequate biomass is essential, as previous work has indicated that a small amount of starting material (i.e., DNA) has adverse impacts on the outcome regardless of sample processing methods [19]. In practice, methods have been adopted as temporary fixes, such as pooling samples from different sites or dates [20] and using wipes instead of swabs as sample collectors [1]. However, these workarounds are not always available [21]. Moreover, systematic evaluation and benchmarking of optimization strategies for metagenomic sample preparation remain largely unexplored.

For challenge 2, propidium monoazide (PMA) is the most widely used viability indicator compatible with molecular techniques. Though intensively optimized [22], its efficacy and applicability in combination with metagenomics are controversial. A semiquantitative systematic evaluation concluded that PMA treatment coupled with 16S rRNA gene amplicon sequencing (PMA-Seq) is reliable when the microbial community is not very complex, while uncertainties increase dramatically with complexity [23]. The uncertainties come from both heterogeneity of microorganisms (e.g., cell envelope structure differences, spore formation) and complexity of the background matrix (e.g., turbidity, salt concentration, dead cell density) [13, 24, 25]. While the microbial communities to be surveilled have their inherent advantage of being low complexity, little is known about the effectiveness of incorporating PMA with multispecies internal standards. To be appropriately rigorous, comparisons are needed relative to standard surveillance that does not consider viability (i.e., no PMA), as well as traditional methods (i.e., cultivation).

For challenge 3, pitfalls of using relative abundances in microbial profiling have been widely indicated. Such pitfalls include but are not limited to lack of unique connections between biological interpretations and experimental observations and unreliable comparisons across samples [14, 26, 27]. Strikingly, mis-selection of analytical tools for relative abundance data could lead to as high as 100% false discovery rates [26]. Besides flow cytometry [28, 29], combing sequencing with quantitative PCR (qPCR) and including internal standards [30] are two major means of making quantitative estimations out of next-generation sequences. In practice, previously reported applications for qPCR include air and dust samples in classrooms [31]; for internal standards, applications include Amazon River plume [32], soil [33], and stool samples [34]. However, comparisons are not yet available between metagenomics coupled with qPCR and with internal standards using low-biomass environmental samples in the immediate vicinity of humans (e.g., healthcare settings).

An additional practical challenge in developing a robust pipeline with metagenomics is how deep one should sequence. While useful in whole genome sequencing, recommendations of coverages expressed by folds of genome sizes (e.g., 15× to 60×) are not readily transferable to metagenomic sequencing (MetaSeq), as reads do not equally distribute across members with substantially different abundances. Nonpareil, a redundancy-based tool, estimates and projects abundance-weighted average coverage for metagenomics (expressed in percentage) [35–37]. This helps reduce erroneous interpretations out of metagenomic results. Yet, expected coverage is still largely unpredictable before sequencing is run. Researchers usually rely on previous experience of similar samples and the available budget to determine the sequencing effort (read size, unit: bp), which may lead to either a coverage too low, thus limiting the extractable information [11], or a waste of resources [1].

**Fig. 1:**
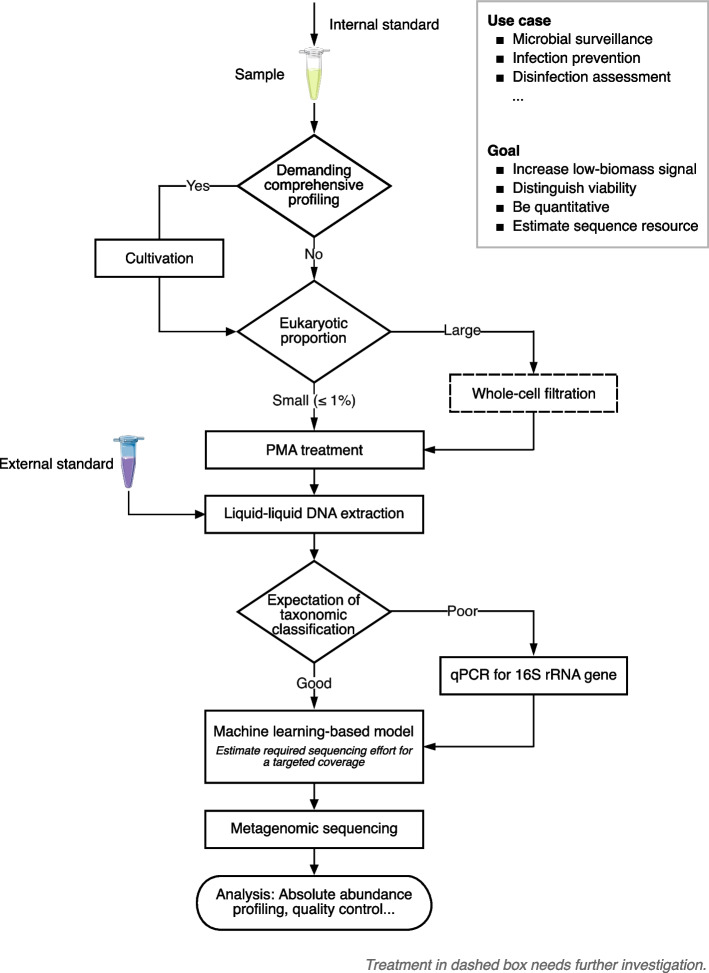
A workflow for metagenomics-based environmental surveillance that is appropriate for low-biomass samples, distinguishes viability, is quantitative, and estimates sequence resources

To address these challenges, we present a workflow for metagenomics-based environmental surveillance that is appropriate for low-biomass samples, distinguishes viability, is quantitative, and estimates sequencing resources (Fig. 1). Liquid-liquid extraction, PMA treatment equipped with internal standards and absolute abundance profiling, qPCR, and a machine learning-based model are the recommended components for the comprehensive workflow, with whole-cell filtration and cultivation as optional accessories.

## Results

### Liquid-liquid extraction improves the power of handling low-biomass samples

To improve DNA yield of low-biomass samples, we first compared 3 categories of extraction methods. Bead beating and heat lysis followed by liquid-liquid extraction were the optimal method, as opposed to widely used column- and magnetic bead-based methods (“Methods,” Additional file 1: Fig. S1) [38, 39]. Notably, no detectable DNA was recovered using Qiagen DNeasy PowerSoil Kit. Supplementary to the recommendation that DNA input ≥ 1 ng for Nextera Flex Library Prep kit, we correlated it to the practical outcome and found that DNA > 11.2 ng corresponded to raw reads > 1e + 05 (Additional file 1: Fig. S1).

**Fig. 2:**
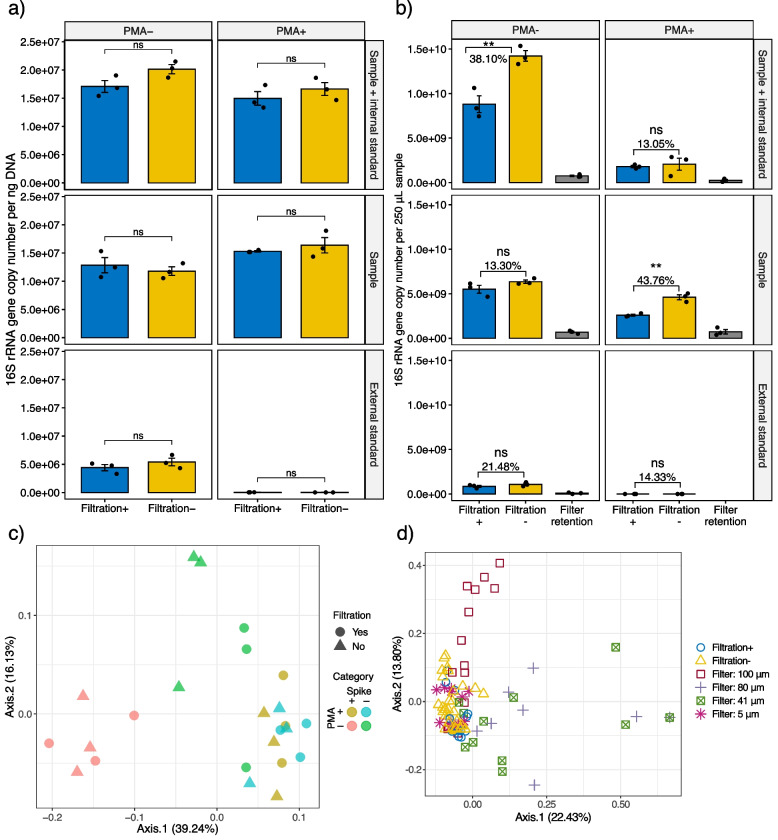
Effects of sequential filtration on hospital-associated surface samples. **a** Bacteria proportion was not significantly increased after filtration according to paired *t*-tests. **b** Biomass of samples with and without filtration as well as retained by filters according to 16S rRNA gene copy number. In **a** and **b**, error bars represent the mean standard error of triplicates. Filter retention includes all biomass captured by 100, 80, 41, and 5 μm filters. Ns and ** are significance codes, representing *p* > 0.05 and 0.001 < *p* ≤ 0.01, respectively. A linear scale was used for both **a** and **b** because for **a**, a linear scale is more conservative than a log scale when no significant difference was concluded; for **b**, linear-scale biomass loss is more informative for metagenomic sequencing. **c** Principal coordinate analysis using Jaccard distance metric among samples with and without filtration. **d** Principal coordinate analysis based on Jaccard distance metric revealed that bacterial profiles retained on 5 μm filters clustered together with liquid samples, while those on 100, 80, and 41 μm filters were away from the major group

In addition to being low biomass, environmental samples of interest are usually in the immediate vicinity of humans and thus often contain eukaryotic cells. These cells may compete with bacteria for sequencing depth, lowering detectable resolution on bacteria, especially for low-abundance members. Collection methods such as swabs and wipes can further recover abiotic debris along with biological materials, to which chemicals potentially interfering with downstream experiments may adsorb [40]. To address these issues, we evaluated the implementation of whole-cell filtration in the workflow. Four filtration steps (100, 80, 41, 5 μm) were conducted in descending order of pore sizes. For our samples, filtration did not exert a significant effect on detected proportions of bacteria (Fig. 2a) or eukaryotic reads (Additional file 1: Fig. S2), according to paired *t*-tests (*p* ≥ 0.05). Considering that the nonbacterial proportion of our samples was relatively small (~1%), filtration appears ineffective (or unnecessary) in increasing the bacterial proportion by excluding eukaryotic cells for samples with similar characteristics. Instead, most of the eukaryotic reads were human-associated and thus able to be removed in silico (Additional file 1: Fig. S2). Moreover, we did not observe an increase in the number of rare taxa post filtration. Nevertheless, filtration did not negatively affect the number of recoverable taxa (Additional file 1: Fig. S3) [41, 42].

As expected, filtration introduced biomass loss of ~13–44%, according to 16S rRNA gene copy number (Fig. 2b). The biomass loss may be compensated by a two-fold concentration, material permitting. Alternatively, the total biomass loss can be reduced in practical applications where one-step filtration is streamlined. Filtration did not impact the overall bacteria composition (Fig. 2c), nor did it change the relative abundances of top abundant taxa (average abundance > 1%) (Additional file 1: Fig. S4). This evidence supports the validity of using filtration to concentrate bacterial samples in sequencing-based experiments for profiling relative abundances. However, the absolute abundances would be affected disproportionately, as the extent of biomass loss varied across samples (Fig. 2b). Therefore, when an absolute metric is of interest, the recovery rate needs to be rigorously measured. Bacteria retention profiles on 5 μm filters were similar to those of the liquid samples. However, bacterial members were not proportionally retained by filters of a larger pore size (100, 80, 41 μm) (Fig. 2d). Hence, treating microbial samples with large pore-size filters may introduce biases, even when relative abundances are used.

Taken together, for samples whose nonbacterial proportion is small (e.g., ~1%), it is unnecessary to incorporate filtration to increase the bacterial fraction. Filtration is valid in concentrating samples. However, for low-biomass samples which are low in both cell density and quantity, biomass loss outweighs the slight increase of bacterial signal. Instead, switching to a high-yield DNA extraction method, such as liquid-liquid extraction, can achieve higher folds of signal improvement (DNA concentration from undetectable to 18.62 ± 1.16 ng/μL).

### PMA and cultivation improve the ability to determine viability

We examined the efficiency of PMA treatment coupled with metagenomic sequencing (PMA-MetaSeq) on hospital-associated surface samples with the ZymoBIOMICS Microbial Community spike-in as the internal standard. The Zymo community consists of 8 bacterial species and 2 yeasts, which presumably will function more comprehensively and accurately regarding bias correction and quality control than a single-species standard [13, 23, 24, 43]. Sequencing outcomes were compared with cultivation results for benchmarking, as cultivation is the gold standard for determining microbial viability.

**Fig. 3:**
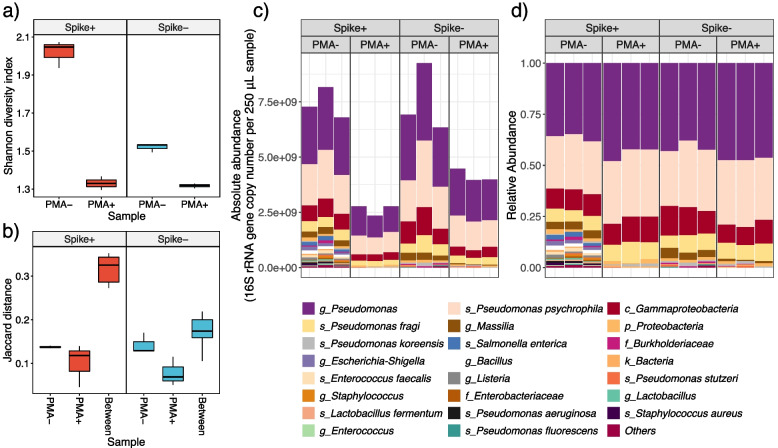
Effects of PMA treatment on hospital-associated surface samples. **a** PMA-treated samples had lower *α* diversity based on Shannon index. **b** Inter distances between paired samples with and without PMA treatment were larger than intra distances within each sample group (based on Jaccard metric). Comparisons of profiling the bacterial composition by **c** absolute abundance and **d** relative abundance

Absolute abundance of samples decreased after PMA treatment, indicating the depletion of nonviable signals (Fig. 3c). This was further supported by the observation that 𝛂 diversity was lower in PMA-treated samples (Fig. 3a), and that inter distances between paired samples were larger than intra distances within each sample group (Jaccard Distance; Fig. 3b). We note that absolute abundance should be used when analyzing sequence data involving viability assessment, as relative abundance profile is likely distorted (Fig. 3 c–d) [33]. Although relative abundance is informative in demonstrating the presence/absence, it neglects the amount of overall biomass and thus may inflate the apparent abundance of even low-abundance organisms. While absolute abundance is more reflective of reality, field trials are necessary to determine whether absolute or relative abundance, or either, can be linked to infection or other clinical outcomes.

We calculated the efficacy of 8 spike-in bacteria [23]. The efficacy should be 1 under ideal conditions, given that the percentage of viable microbes in the Zymo community is negligible (Additional file 1: Fig. S5). The efficacy equaled 1 for all taxa, suggesting that PMA treatment is effective in low-biomass samples regardless of taxonomy. This conclusion is partly consistent with our previous evaluation of PMA-Seq where *E. coli* was spiked in [23].

**Fig. 4:**
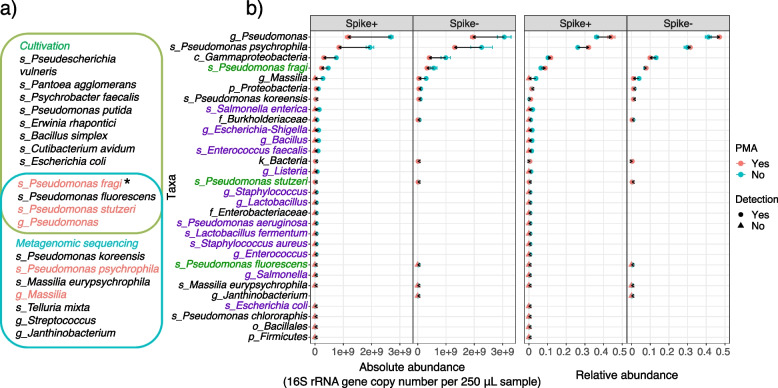
Performance of cultivation and PMA-MetaSeq in viability distinction of hospital-associated surface samples. **a** Venn diagram showing the detected taxa by cultivation and MetaSeq. Taxa detected by PMA-MetaSeq are color coded in red. *Pseudomonas fragi* was also detected in the genome-centric approach and is marked with an asterisk. **b** The abundance change of all taxa detected under the framework of absolute abundance and relative abundance. Taxa in the theoretical composition of the internal standard and recovered in cultivation are color coded in purple and green, respectively. The *Y*-axis follows a descending order of the average abundance across samples. Error bars represent the mean standard error of triplicates

Focusing on specific taxa (Fig. 4b), we observed occasions of a complete depletion for high-absolute-abundance taxa and retention for low-absolute-abundance taxa, suggesting an effective viability distinction. Relative abundance for some taxa increased after PMA treatment (*g_Pseudomonas*, *s_Pseudomonas psychrophila*, *c_Gammaproteobacteria*, *s_Pseudomonas fragi*, *s_Pseudomonas koreensis*, *k_Bacteria*), while all taxa showed a decrease in absolute abundance. This indicates that PMA treatment may increase the ability to detect taxa with majoritarily viable populations. We did not detect new taxa that were previously undetectable in PMA-treated samples. However, if nonviable microbes are not of interest, treating samples with PMA will improve the detection power for the overall community with comparable sequencing resources (Fig. 6c), as it reduced the overall 𝛂 diversity (Fig. 3a).

Cross-referencing between cultivation and PMA-MetaSeq was greatly impeded by their inherent limitations (e.g., detection limit for MetaSeq, biases with bioinformatics; viable but non-culturable cells for cultivation). Even for PMA-untreated samples, cultivation and MetaSeq only agreed with each other on a small number of taxa (Fig. 4a). Among the 3 viable taxa confirmed by cultivation, viability of *s_Pseudomonas fragi* and *s_Pseudomonas stutzeri* was reflected by PMA-MetaSeq, while *s_Pseudomonas fluorescens* became undetectable after PMA treatment. This might imply over-depletion but could also be because its abundance went below the detection limit of MetaSeq. As indicated by Barbau-Piednoir et al., less-abundant taxa were more likely to be eliminated (to undetectable) by PMA treatment [44]. This is consistent with our observation, as the abundance of *s_Pseudomonas fluorescens* was the smallest among the cultivation-confirmed taxa. Thus, for low-abundance taxa, cultivation could serve as a supplement to sequence-based viability assessment techniques, as a small unintentional removal of viable cells may lead to a large presence/absence difference. Moreover, incorporating cultivation can expand the detection spectrum in general, and particularly for low-abundance taxa, due to MetaSeq’s restrictions such as detection limit and failure to distinguish closely related taxa.

Collectively, we emphasize the importance of using absolute abundance and demonstrate a successful application of multispecies internal standards in PMA-MetaSeq. PMA is effective in low biomass samples and can improve the detection power by eliminating irrelevant signals. Cultivation remains a valuable supplement to sequence-based techniques for capturing a comprehensive picture.

### Poor taxonomic classification is a major hurdle for internal standards in quantitative metagenomics

Quantifying metagenomics-based abundances using internal standards has substantial benefits. Theoretically, addition of internal standards could compensate for errors resulting from nonquantitative steps [13]. *E. coli* is one of the most used spike-in strains, in part because it is well-studied and easy to recover in sequencing [23]. However, ideally, we want the internal standard to contain a set of diverse taxa, so that it well represents the diversity in microbial communities. We investigated the performance of the Zymo community as the internal standard for hospital-associated environmental samples, along with qPCR for the 16S rRNA gene.

Unfortunately, the efficiency of implementing the Zymo standard in quantitative metagenomics was drastically impeded by the limited resolution of taxonomic classification. We tried two approaches: Metaxa2 [45–47] coupled with the SILVA 132 SSU database [48, 49] and MetaPhlAn3 [50, 51], which uses a collection of marker genes. The taxonomic resolution varied substantially across different taxa. For samples containing only the Zymo standard, 85% of the small subunit rRNA reads were attributable by Metaxa2, while only 48.14% of the metagenomes were recognized by MetaPhlAn3. Within the attributable portion, MetaPhlAn3 performed better regarding specificity; all reads were classified at the species level, while Metaxa2 retained a decent amount of information at higher levels, with the ratio of genus/family-level and species-level classifications ranging from 0.18 to 11.28.

Foreseeably, this issue will be alleviated as reference databases and taxonomic assignment tools continue to advance. Currently, advantages of internal standards are mainly reflected when species-level identification is the major focus. For instance, clinical samples usually target pathogenic species whose core pangenomes are relatively well represented in databases. In this case, the biases from uneven representation of species can also be corrected based on the performance of closely related internal-standard species. However, if information at genus or higher levels is of consideration, internal-standard techniques become non-applicable, as we are not able to distinguish internal-standard taxa from other species within the same genus (or at higher levels), which is the basis of making calculations and corrections. Coupling with qPCR, instead, is more appropriate (Fig. 3c). Environmental communities are typical examples where coupling with qPCR stands out because environmental microorganisms are not usually well represented at the species level. Of 87 samples in our study, strikingly, MetaPhlAn3 only recognized an average of 19.68% of the metagenome at the species level. The classification rate slightly increased to 38.40% using Metaxa2, which substantially improved to 87.24% when genus level was included.

### Accessible sample features can predict required sequencing effort

To enable more informed decision-making before MetaSeq, we conducted a quasi-meta-analysis, using the limited number of existing hospital-related environmental metagenomics studies [1, 5, 20, 52–54]. We recruited 956 shotgun samples (874 from 6 previous studies and 82 from this study) (Additional file 2). Using these data, we linked accessible features (e.g., location, building, sampling method) to the required sequencing effort given a targeted coverage, leveraging machine learning-based models and Nonpareil (Fig. 6a).

**Fig. 5:**
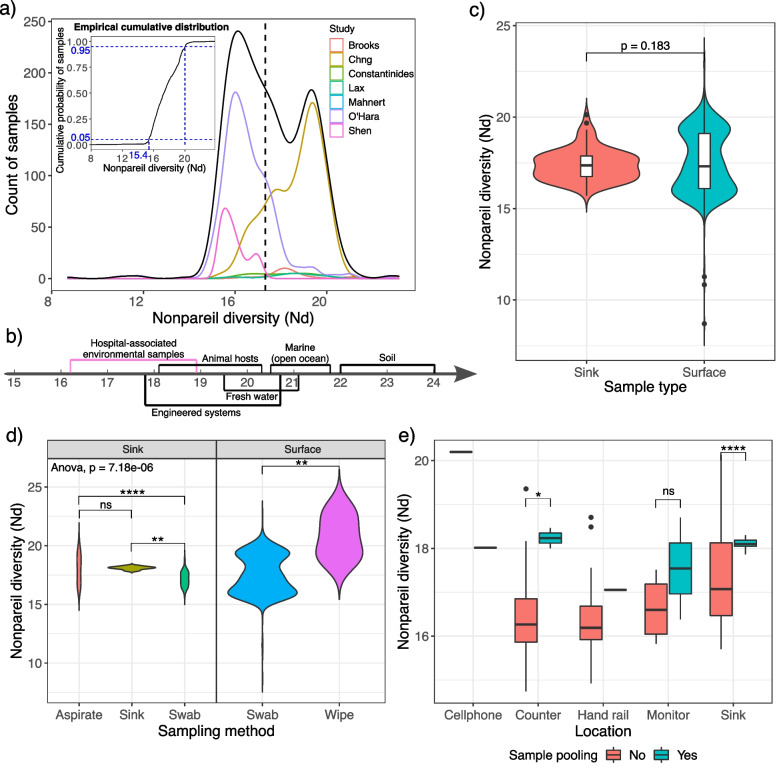
Relationships between Nonpareil diversity and metadata features for hospital-associated surface samples. **a** Overall distribution of Nonpareil diversity (black) and distributions for individual studies. **b** Interquartile range of Nonpareil diversity for microbiome samples from different environments. This study is color coded in orchid. Effects of **c** sample type, **d** sampling method, and **e** sample pooling on Nonpareil diversity. Significance codes are as follows: *p* > 0.05 (ns), 0.01 < *p* ≤ 0.05 (*), 0.001 < *p* ≤ 0.01 (**), *p* ≤ 0.001 (***)

**Fig. 6:**
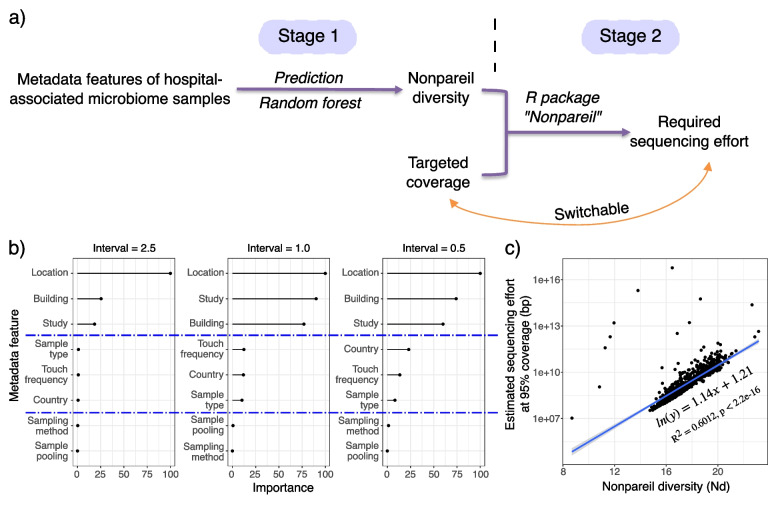
Required sequencing effort can be predicted by accessible sample features and targeted coverage. **a** Workflow of making the prediction. **b** Variable importance rankings by random forest. **c** The natural log of estimated sequencing effort at 95% coverage is linearly correlated with Nonpareil diversity

Relationships between Nonpareil diversity (Nd, unit: log-bp) and metadata features were first explored (Fig. 6a: stage 1). Nd is an index measuring the complexity of a microbial community regarding “sequence space,” which correlates with classic bin-based diversity indices (e.g., Shannon index) for bacteria [35, 37]. Though not passing the normality test (Shapiro-Wilk test, *p* = 3.338e-16) [55], normal distribution was still the best-fit distribution of our dataset, followed by logistic distribution, upon investigation by Cullen and Frey Graph and R package “fitdistrplus” (Fig. 5a, Additional file 1: Fig. S6). Presumably, the deviation from normality will decrease as sample sizes increase. For 90% of samples, Nd was within 2 orders of magnitude (15.4–20.0, natural log scale), suggesting a common range for hospital-associated environmental samples, which is valuable for reference when designing future studies. Notably, this Nd level was among the lowest across 6 different environments including animal hosts, fresh water, and soil (Fig. 5b) [37].

We further examined the influences of sample type (sink versus surface), sampling method and sample pooling on Nd. No significant difference was observed between sink and surface samples (Fig. 5c). Within sink samples, Nd was significantly different across sampling methods (ANOVA, *p* = 7.18e-06). Specifically, samples collected by swabs seemed to have a smaller diversity than those by the other methods (Tukey’s post hoc test; samples without a clear collection method stated in the original paper were assigned as “sink”). Note that even though sink samples are generally from the same location, the confounding effects introduced by sub-locations (e.g., sink basin, pipe edge, P-trap) cannot be ruled out. Similarly, within surface samples, though Nd of wipes was significantly larger than that of swabs (unpaired *t*-test), confounding effects remain (e.g., researchers tend to use wipes for large-area and high-biomass locations, like floors, which often contain more diverse communities) (Fig. 5d). Though weak, we noticed a trend of diversity increase after sample pooling (Fig. 5e), raising the alarm that more caution should be taken when increasing biomass by pooling samples. The practice of sample pooling assumes that the pooled samples share some core features, whose biomass will be increased past the detection limit. This may be true of certain sample types, e.g., host-associated microbiomes, but is unlikely to be true of built-environment samples that lack a conserved core [56]. Further investigations are needed should more data become available, as the sample size was quite limited for some groups (e.g., n(pooled monitor) = 2, n(not-pooled monitor) = 4). In the interim, we recommend seeking other methods, such as a high-yield DNA extraction, before resorting to sample pooling, as the resulting sample characteristics may be different from individual samples.

To further harness the reference potential of Nd, we built models to predict Nd from metadata features based on machine learning algorithms. Eight predictor variables (location, building, study, country, touch frequency, sample type, sampling method, sample pooling) were included based on data availability, MIxS-BE standards, and previous experience (Additional file 2) [53, 57, 58]. Nd, the response variable, was first converted from a numerical variable to a nominal variable. Three conversion schemes were tried, with the intervals being 2.5, 1.0, and 0.5 (number of categories being 2, 5, 11, respectively). Random sampling was adopted to split the entire dataset into training and testing datasets at the ratio of 4:1. Implementing repeated cross-validation (5 folds, 5 times) on the training dataset, 9 algorithms were examined to optimize the classification performance, including random forest, stochastic gradient boosting, and support vector machines. Algorithms were evaluated according to 4 metrics (area under curve, Kappa, and balanced accuracy on both training and testing datasets) [59]. Overall, no difference was observed among the tested algorithms. Random forest was selected due to its slightly better performance from a holistic perspective and capability of ranking the predictor variables.

The model accuracy positively correlated with the interval size. At 2.5, the accuracy on the training dataset was as high as 87.69% and slightly lower on the testing dataset (82.60%). The accuracy dropped as the classification demand rose. The mean balanced accuracy on the testing dataset was 64.08% and 61.38% when intervals were 1.0 and 0.5, respectively. Considering that Nd was converted from a continuous variable, we examined the misclassifications and found that most of them fell into nearby categories. We thus calculated the mean balanced accuracy ± 1 category and observed a substantial improvement. Specifically, mean balanced accuracy of 87.06% and 77.17% can be achieved for 5- and 11-category classifications, respectively. Considering the sparsity of the currently available dataset and the challenge of multiclass classification, this model demonstrated a reasonable degree of accuracy, which should improve as sample sizes and available features grow.

The variable importance ranking generated by random forest separated the predictor variables into 3 groups (Fig. 6b). Location, building, and study were the top 3 variables with the highest importance, followed by country, touch frequency, and sample type, while sample pooling and sampling method hardly impacted the classification. Groupwise, this ranking was generally consistent with the explanatory power described by linear regression (Additional file 1: Fig. S7) [58]. That “study” ranked as one of the most important variables indicated the existence of biases towards individual studies in the current dataset (e.g., “batch effects” related to respective sampling, processing, sequencing, and analysis), which was also observed by a previous meta-analysis of indoor microbiota [58]. Interestingly, despite its high importance, the model performance had almost no drop after excluding “study” (> 95% for all conversion schemes), justifying making predictions without involving artificial metadata features like “study.” It is worth noting that importance of the other variables (e.g., building, country) was raised after this exclusion (Additional file 1: Fig. S8). To find the features necessary for making a comparably accurate prediction, we further examined the performance of models after gradually reducing the number of predictor variables and found that using only 2, “location” and “building,” the new model achieved 95% accuracy regardless of interval sizes tested.

With Nd and metadata features connected, the required sequencing effort at a targeted coverage was then inferred (Fig. 6a: stage 2). Upon fitting the data, we revealed a linear relationship between the natural log of estimated sequencing effort at 95% coverage (ln(LRstar)) and Nd, with the equation being ln(LRstar) = 1.14 × Nd + 1.21 (adjusted R-squared = 0.6012, *p* < 2.2e-16) (Fig. 6c). This is theoretically backed up by previous findings that sequencing effort depends on the diversity level and the genome size, and that the latter can be ignored for most microbial communities, particularly bacterial communities, since the differences in genome size are usually no more than one order of magnitude [35]. Instructions to make calculations between sequencing effort and other coverage levels are provided at https://github.com/jxshen311/workflow_metagenomic_environmental_surveillance/tree/main/nonpareil/example_SeqEffort%26Coverage.

## Discussion

Although sequence-based environmental surveillance of microbial communities for better management of public health has been appealed for and utilized, best practices of the workflow have not been systematically studied to ensure proper interpretations of sequencing results to aid in infection risk assessment [13, 14]. This study introduces a well-structured and informed metagenomics-based workflow towards the goal of being appropriate for low-biomass, viability, quantification, and resource estimation. We recommend adopting liquid-liquid extraction to improve DNA yield and only incorporating whole-cell filtration when nonbacterial proportion is large. Despite its imperfection, we suggest including PMA treatment, and involving cultivation when demanding comprehensive profiling. We further recommend integrating internal standards for quantification, and additionally qPCR when we expect poor taxonomic classification. We also introduce a machine learning-based model to predict required sequencing effort from accessible sample features. The model helps make full use of sequencing resources and achieve desired outcomes.

While using realistic samples in testing simulates conditions the workflow may face in practical applications, it comes with side effects. Our aggregation sample had a small fraction of nonbacterial organisms (~1%). Thus, the conclusion that whole-cell filtration does not increase the bacterial proportion and signal of rare taxa to a statistically significant degree is probably only applicable to samples with similar characteristics, representing 84.90% among the 874 samples from hospital-related environmental studies used in the quasi-meta-analysis [1, 5, 20, 52–54]. However, a few samples did contain a decent proportion of eukaryotes. Specifically, 132 samples harbored more than 1% eukaryotic reads, and strikingly, more than half reads were attributed to eukaryotes for 20 samples. Moreover, samples collected from high-touch surfaces were more likely to have higher proportions of eukaryotes than low-touch surfaces and sinks. Of the 104 sink samples, the maximum percentage of eukaryotes was 0.1%. Therefore, filtration is probably unnecessary for most environmental samples (especially sink samples) and may be beneficial for part of high-touch surface samples (Additional file 1: Fig. S9).

Despite being semiquantitative and entailing considerable uncertainty, involving PMA takes us a step closer to understanding viability, particularly for low biomass samples whose complexity is also relatively low [23]. Notably, the overall uncertainty comes not only from PMA treatment but also from the metagenomics pipeline itself, such as biases from DNA extraction kits and taxonomic assignment tools [16, 60]. In addition to PMA, alternative metrics have been proposed, including methods based on RNA (reflects active transcription), peak-to-trough ratio (PTR) (reflects active replication), and nuclease digestion (e.g., benzonase). As stated in a systematic evaluation, while 16S rRNA transcript-based amplicon sequencing semi-quantified viability of synthetically constructed simple communities (*Escherichia coli* and *Streptococcus sanguinis*), it is inappropriate for realistic complex communities [61]. PTR has been demonstrated as an efficacious metric to estimate microbial growth rates in both human (e.g., skin, fecal) and environmental (e.g., marine, sludge) datasets by several studies [62–65]. However, a study based on freshly collected marine prokaryotes raised concerns as they observed poor correlations between PTR and growth rates for most marine bacterial populations (r ~−0.26–0.08), except for the rapidly growing γ-Proteobacteria (r ~0.63–0.92) [66].

Some overlap exists between methods for viability determination and those for depleting eukaryotic DNA. For example, osmotic lysis followed by PMA treatment is recommended to remove human DNA in saliva samples [67]. However, recommended methods depend on the sample type. PMA is not recommended for sputum samples, where nuclease-based methods (e.g., digest with benzonase) showed an equal or better performance [68]. Benzonase has also been applied to skin microbiome samples with desired outcomes [69]. In general, factors impacting method performance include percentage and composition (e.g., extracellular DNA, DNA in largely lysed or partially compromised cells) of targets to be removed (i.e., eukaryotes and dead bacteria), as well as characteristics of background matrix (e.g., viscosity). For instance, saliva and sputum consistently contain ≳ 90% human DNA [67, 68], while this percentage is very diverse for hospital-associated environmental samples (Additional file 1: Fig. S9). Filtration failed to exclude human DNA in saliva likely because extracellular DNA was the dominant component rather than cells [67]. For sputum samples where cells are lysed and DNA is no longer protected, nucleases might be quite effective in depleting extracellular DNA, whereas PMA efficacy could be hindered by the viscosity of the matrix) [68]. In contrast, in environments where cells gradually decay due to harsh conditions (e.g., desiccation), more DNA attributable to dead cells would still have a partially compromised membrane; PMA, as a small molecule, may be more effective in penetrating the damaged cell membrane and depleting the DNA. For eukaryotic depletion, it may be beneficial to further unravel the underlying mechanisms influencing the efficacy of different methods in different sample types and characteristics.

Nevertheless, for viability assessment, instead of focusing on this viable/dead dichotomy, perhaps more critically, we should keep in mind that “viability” is rather an intermediate or methodological term, linking surveillance results to questions of interest (e.g., which bacteria are infectious) [13]. In the future, it is worth exploring whether the concept “viability” in the context of interest is closer to intact cell membrane, active transcription, or active replication. Moreover, rather than optimize one single metric, integration of multiple methods has been proposed (e.g., using multi-omics techniques) [23]. Pursuing viability profiles using orthogonal methods would plausibly enable a more comprehensive understanding, but the cost-benefit ratio may be considerably high for multi-omics techniques. Integrating with cultivation, instead, provides an affordable alternative. Notwithstanding, it remains to be investigated how to properly interpret results generated by a combination of methods, as inconsistencies between disparate methods are common.

We applied multi-taxa internal standards and calculated PMA efficacy of spike-in taxa based on a reasonable assumption that the percentage of viable microbes in the Zymo community is negligible, resulting in a theoretical value of 1 (Additional file 1: Fig. S5). While this internal standard can strongly reflect incomplete suppression of nonviable signals, potential toxicity of PMA might be underrepresented. Although no toxicity was observed at the PMA dose of our protocol in validation (Additional file 1: Fig. S10), a customized internal-standard mixture featuring 0.5 as the designed PMA efficacy would be ideal for future studies [23]. As opposed to purchasing commercial products, we recommend utilizing the Zymo community as a reference for the taxonomic composition and constructing the mixture with live cultures in real time, because viability (or membrane integrity when PMA is used) is difficult to maintain in manufacturing, shipping, and storage.

Continuous advancement of internal standards for quality control, as well as quantification and other features, is still one of the major hotspots in method optimization. A suitable internal standard should well balance representation and recognizability. Good representation means that the workflow impacts the spike-in and targeted microbes comparably (because of their similarity). Good recognizability means that the spike-in can be easily distinguished from the targets. In this study, the Zymo community was selected due in large part to its representation, as it spans broadly the phylogenetic tree. Previous studies have selected internal standards based on a similar principle. For instance, the Zymo community and a 10-species mock community were chosen for gastrointestinal and stool samples, respectively [70, 71]. Peroxide-killed *Campylobacter sputorum* was used to quantify viable thermotolerant *Campylobacter* [72]*.* These internal standards are prone to be confounded with targets, thus posing challenges for bioinformatics to accurately identify and quantify taxa. To obtain good recognizability, exotic materials are sought. In the aforementioned example, the researchers chose 10 species that were generally absent from the stool of healthy individuals. The same criterion was followed by another gut microbiome study in which microbes from hypersaline environments, soil, and plants were utilized [34], as well as a study on Amazon River plume to which genomic DNA from *Thermus thermophilus* HB8 was applied [32]. Finding a completely exotic species is more challenging for environmental surveillance whose subjects are influenced by both human and environmental activities. As a potential solution, artificial DNA have been developed to ensure differentiation from the targets. Previous reports included sets of synthetic DNA, 16S rRNA genes, and chimeric DNA fragments, implemented in different venues of metagenomic and amplicon sequencing [33, 73, 74]. However, whether these exogenous (or even artificial) standards’ behavior resembles that of the targets remains questionable. By and large, more systematic evaluation and optimization are needed to foster the development of internal-standard techniques that better balance representation and recognizability or at least make their pros and cons quantitatively accessible, both in general and for specific contexts. For example, it will be beneficial to conduct data-driven meta-analyses and curate databases to further inform the field.

Though the classification models performed well from a practical perspective, their accuracy with small intervals still merits improvement. Building a hierarchical classification model might be beneficial, as we observed a drastic increase in the accuracy when the interval size was enlarged. It is also likely that the available dataset is not good enough to train a model with very high accuracy. For example, there is clear evidence that the data were biased by the disparate sample sizes between studies. Moreover, we only managed to collect 7 common metadata features (excluding “study”) without involving a substantial number of missing values, which raises the question of whether what we achieved has already reached the theoretical plateau of explanatory power of these features. If this is the case, standardized reporting of more high-quality metadata should be further promoted. Additionally, since normal distribution was the best-fit distribution of the current dataset, with seemingly missing pieces in the middle (Fig. 5a), fitting data into known distributions may be more explanatory as large sizes of data become accessible.

## Conclusions

This study presents an improved workflow towards accurate and robust healthcare environmental surveillance using metagenomics. The workflow is appropriate for low-biomass samples, distinguishes viability, is quantitative, and enables estimation of necessary sequencing resources. We recommend liquid-liquid extraction, propidium monoazide treatment coupled with internal standards and absolute abundance profiling (e.g., using qPCR), and a machine learning-based model for sequencing depth calculation. In addition, whole-cell filtration and cultivation may be valuable under particular circumstances.

This metagenomics-based environmental surveillance workflow is particularly useful in infection prevention and disinfection assessment. Although we focus on microbial surveillance of built environments, especially hospital-associated surfaces, the workflow developed in this study can be adapted to other contexts with similar characteristics. For example, the multifaceted lessons learned from this study will benefit the continuing development of microbiome-based clinical testings from body sites (e.g., skin), such as methods to increase low-biomass signals and determine viability [12]. Moreover, the experience gained in overcoming challenges unique to environmental microbiomes (e.g., quantitative metagenomics with poor taxonomic classifications) is also useful to studies on other environments, such as wastewater and air.

## Methods

### Sample collection, aggregation, and cultivation

We collected 120 surface swabs from the 28-bed medical intensive care unit (MICU) at Rush University Medical Center (RUMC) in October 2018. RUMC is a 720-bed tertiary care teaching hospital in Chicago, IL, USA. Samples were collected from door sills, computer keyboards, light switches, nurse call buttons, and bed rails in 13 single-bed patient rooms, as well as door sills in 4 medication rooms, 2 public restrooms, 1 staff-only restroom, and the communicating space of MICU (Additional file 3). Weighted mean area of sampled surfaces was 216 cm^2^. Patient rooms were selected to keep a relatively balanced number for both contact isolation and noncontact isolation rooms. Healthcare providers and visitors entering contact isolation rooms are required to wear gowns and gloves, which may reduce transmissions via contaminated healthcare providers. Room temperature and relative humidity were documented during the collection, which varied slightly across rooms, with the average being 23.8 °C and 45%, respectively. Each sample was collected by 3 COPAN Nylon Flocked Swabs (Copan Diagnostics, Murrieta, CA, USA) and 1.5 mL phosphate-buffered saline with 0.02% Tween 80 (PBST) and stored at 4 °C for up to 24 h prior to extraction, aggregation, and cultivation [53, 75]. Swabs were extracted and aggregated to create a representative microbiome sample [56, 75, 76]. Aliquots of this aggregation sample were then subjected to different processing methods (i.e., several DNA extraction methods, microbial community standard spike-in, PMA treatment and whole-cell filtration) to find best practices of the workflow (Additional file 1: Fig. S11).

To capture a large fraction of the indoor microbiome diversity, we cultured the samples with 4 different media: tryptic soy agar (TSA), Reasoner’s 2A agar (R2A), 0.1 strength R2A at 25 °C, and blood agar (BA) at 37 °C, all supplemented with 4 mg/L itraconazole [75]. This resulted in 233 cultivable isolates. All colonies that could be individually picked or purified were subject to taxonomic identification by matrix-assisted laser desorption/ionization time-of-flight mass spectrometry (MALDI-TOF MS) using the VITEK® MS Mass spectrometry microbial identification system (BioMerieux, Marcy-l'Étoile, France) and the VITEK MS V3.2 FDA 510(k) cleared database. Among the 233 isolates, 201 were identified. It is important to note that because multiple media types were used, the number of isolates for each species identified does not represent the relative abundance of this species in the sample, as some species may have grown on multiple media.

### Standard addition, PMA treatment, and whole-cell filtration

All treatments were done in triplicate, including cultivation.

#### Standard addition

Aliquots were snap frozen and stored at −80 °C until further processing to maximize the integrity of samples and avoid degradation resulting from long-term storage at 4 °C [76, 77]. Samples were thawed at 4 °C prior to treatments. ZymoBIOMICS Microbial Community Standard (Zymo Research, Irvine, CA, USA) was used as both the internal standard and the external standard. As the internal standard, 6.50 μL Zymo community was spiked into 1 mL aggregate sample, following the criterion that DNA of the species with the highest abundance in the Zymo community approximates 1% of the total DNA in the aggregate sample [32, 78]. As the external standard, aliquots of the Zymo community were run in parallel with aggregate samples throughout the workflow to assure its performance.

#### PMA treatment

Following standard addition, PMA treatment (Biotium, Fremont, CA, USA) with an optimized protocol was applied to half of the samples within each group [22, 24, 25, 79, 80]. The protocol was first validated by reproducing the work of Nocker et al. (2006) using *Escherichia coli* (ATCC 8739) as model strain (Additional file 1: Fig. S10). *E. coli* was grown to the exponential phase. The culture was then split into two aliquots, one of which was killed by heat inactivation at 95 °C for 7 min in Eppendorf ThermoMixer shaking at 400 rpm for homogenized heating. After cooling to room temperature, live and heat-killed aliquots were mixed following the same ratios as in Nocker et al. (2006), yielding samples of 6 different expected live cell ratios. Viability of both live and heat-killed cultures was confirmed by spread plating onto TSA and incubating at 37 °C overnight. Half of each constructed sample underwent PMA treatment. The results were evaluated by both DNA concentration ratio quantified by Quant-iT™ PicoGreen™ dsDNA Assay (ThermoFisher, Waltham, MA, USA) and copy number ratio by qPCR with 16S universal primers (341F and 534R) (Additional file 1: Fig. S10). Briefly, a final concentration of 25 μM PMA was used, and several steps were conducted to ensure the consistency across samples and minimize nonspecific reactions between PMA and random sample components, including (1) adding PMA to tube caps and inverting all tubes simultaneously, (2) working under red light, and (3) protecting samples from light as much as possible before the light activation step in the PMA-Lite™ device (Biotium, Fremont, CA, USA). An aliquot of samples for each replicate was preserved at −80 °C until DNA extraction, with the rest stored at 4 °C for downstream filtration.

#### Whole-cell filtration

Whole-cell filtration was conducted using EMD Millipore 25 mm Glass Vacuum Filter kit (MilliporeSigma, Burlington, MA, USA), 125 mL filter flask, and Gemini vacuum pump in a biosafety cabinet following aseptic techniques (Additional file 1: Fig. S12). Notably, autoclaved tweezers were used to avoid possible contaminations from touching sensitive parts of the setup. Samples were filtered by 100 μm nylon membrane, followed by 80 μm and 41 μm nylon membranes and 5 μm PVDF membrane (MilliporeSigma, Burlington, MA, USA). A total of 1 mL PBS was added to the falcon tube and flask at each step to increase the sample recovery by rinsing the inner wall. The filtered samples were then subjected to 3-fold (relative to the volume before filtration) vacuum concentration with an Eppendorf Vacufuge plus. Filtered liquid samples and filter papers were preserved at −80 °C until DNA extraction. To increase the extraction efficiency from filter papers, we compared (1) cutting them with scissors into 9 pieces, (2) grinding them with metal spatula after snap freezing in liquid nitrogen, and (3) directly putting the whole filter paper into the preservation tube. We finally selected the third option as this was the most operationally feasible way without high risk of contamination.

### Negative controls

To combat the susceptibility of low-biomass samples to contamination, we included 4 types of negative controls along the workflow, namely, 6 negative field controls, 6 negative media controls, 12 negative filter controls, and 7 negative kit controls [13, 15, 16, 58]. The negative controls were processed in parallel with the surface samples, including metagenomic sequencing and bioinformatic analysis.

Negative field controls were collected exactly the same as surface samples, except that the swabs were exposed to the air without contacting targeted surfaces. One negative field control was collected at the beginning and the end of each sampling session. Two negative media controls were included in each sampling session, which were unopened media with swabs from the same lot. Each collected control was split into triplicate and processed along with samples [53, 81]. Negative filter controls were included in triplicate for each pore size by letting sterile PBST flow through the vacuum filtration system attached with blank filter papers. Additionally, 7 negative kit controls were processed across batches of DNA extractions.

### DNA extraction, qPCR, and metagenomic sequencing

To ensure enough DNA recovery, we performed an initial optimization on a separate set of surface swab samples collected from the same MICU prior to working with the aggregate sample (Additional file 1: Fig. S1). Column-based methods were first tried due to its widespread usage in the field. We examined Qiagen DNeasy PowerSoil Kit with standard protocol and a modified version by (1) changing from vortex lysis to bead-beating lysis, (2) introducing heat incubation after bead beating, and (3) adding 50 μL water each time for twice in total at the elution step. DNA yields of both were below the limit of detection. Liquid-liquid extractions were performed afterwards for their high-yielding potentials. Phenol-chloroform extraction resulted in the highest yield (186.27 ± 55.51 ng/μL by NanoDrop), but the purity indicated by 260/280 was not acceptable (1.36 ± 0.03). Lucigen MasterPure™ Complete DNA and RNA Purification Kit also resulted in high yields when coupled with bead beating and heat lysis and better purity than phenol-chloroform extraction (260/280 1.62 ± 0.02). We attempted to improve the purity using the Agencourt AMPure XP PCR Purification kit. However, we did not see a purity increase (260/280 1.62 ± 0.03) and incurred a 64.70% DNA yield drop. Based on the above tests, we noticed that methods involving columns (Qiagen PowerSoil) or magnetic beads (Agencourt AMPure) greatly decreased the DNA yield. Because the primary concern for surface samples is low biomass, increasing DNA yield is considered more critical than bringing 260/280 to the desired range of 1.8−2.0. Therefore, the Lucigen MasterPure™ Complete DNA and RNA Purification Kit with the adapted protocol was chosen for all subsequent analyses [82]. Samples were thawed at 4 °C prior to DNA extraction, and DNA concentrations were quantified by Quant-iT™ PicoGreen™ dsDNA Assay [83].

V3 region of the 16S rRNA gene was amplified in qPCR using universal primers (341F: 5′-CCT ACG GGA GGC AGC AG-3′, 543R: 5′-ATT ACC GCG GCT GCT GGC A-3′) [40]. The 20 μL reaction mixture consisted of 10 μL PowerUp™ SYBR™ Green Master Mix (Applied Biosystems), 0.6 μL forward primer (10 μM), 0.6 μL reverse primer (10 μM), 5.0 μL DNA templates (pre-diluted if necessary), and 3.8 μL nuclease-free water. The reaction was run in technical triplicate on a QuantStudio 3 Real-Time PCR System (Applied Biosystems) with an initial denaturation step at 95 °C for 2 min, followed by 40 amplification cycles (95 °C, 15 s; 56 °C, 15 s; 72 °C, 1 min) and a melting curve stage (95 °C, 15 s; 60 °C, 1 min; 95 °C, 15 s). No-template control and 5~8 standards were included in each batch to generate the standard curve (efficiency > 90%; *R*^2^ > 0.99). Plasmid DNA constructed by TOPO™ TA Cloning™ Kit (Invitrogen, Waltham, MA, USA) was used as standards.

Extracted DNA was shipped on dry ice to the UMICH Microbiome Core (Ann Arbor, MI, USA) for library preparation using Nextera™ DNA Flex Library Prep Kit and paired-end 250-bp shotgun metagenomic sequencing on an Illumina MiSeq platform (MiSeq Reagent Kit v2). Libraries were normalized at equal molarity in a 4 nM final concentration pool before sequencing, and for samples without enough DNA (e.g., negative controls), all available materials were used.

### Data analysis

#### Sequence data processing

KneadData (v0.6.1) was first used to clean the shotgun sequences with default parameters. Reads present in the human reference database (hg37_and_human_contamination) and negative controls were filtered out. Metaxa2 (v2.2) [45–47] coupled with SILVA 132 SSU database [49, 84] was chosen to generate taxonomic profiles after comparing it with MetaPhlAn2 (v2.6.0) [85] and MetaPhlAn3 (v3.0.7) [50, 51]. The evaluation was conducted based on their performance on external standards and cross validation with cultivation results for untreated aggregate samples. Default parameters were used for all three tools. MetaPhlAn3 was ruled out mainly because it only generates marker genes at the species level, and an average of 80.32% metagenome was deemed unknown for our samples. For external standards, both Metaxa2 and MetaPhlAn2 recognized all 8 bacteria species demonstrated in the theoretical composition, but MetaPhlAn2 failed to classify the 2 eukaryotic species. Moreover, it did not classify *Pseudomonas fluorescens* and barely classified *Pseudomonas stutzeri* from aggregate samples, while Metaxa2 recognized both. Though Metaxa2 included a few spurious taxa, all can be eliminated by removing singletons. Since the primary goal of this study was to compare techniques and recommend best practices, sensitivity outweighed specificity. Therefore, Metaxa2 was selected, and singletons were removed for downstream analyses. Taxa were labeled to the lowest classifiable level, with species level as the ultimate target [23]. Metagenomic sequencing coverage for all samples was estimated by Nonpareil (v3.303) under kmer mode using default settings [35, 37].

#### Comparison between gene- and genome-centric approaches

An evaluation of genome-centric analysis was based on 9 untreated aggregate samples (Additional file 4). We first compared MEGAHIT (v1.0.6.1) [86] and metaSPAdes (v3.14.1) [87] and selected metaSPAdes as the assembly tool because it produced better-quality contigs (i.e., longer and fewer contigs, higher N50) as assessed using QUAST (v4.4) [88]. MetaBAT2 (v1.7) [89] was then used to bin the contigs and reconstruct genomes, with bin quality checked by CheckM (v1.0.18) [90]. Each sample yielded a single high-quality bin (completeness > 92%, contamination < 2%). Taxonomy of the bins was subsequently assigned using GTDB-Tk (v1.7.0) [91]. All the 9 metagenome-assembled genomes were classified as *Pseudomonas fragi*. The genome-centric approach thus represents the least sensitive method compared with cultivation and gene-centric metagenomics, especially for less abundant taxa. Because the genome-centric method only detected 1 taxon, while cultivation and gene-centric metagenomics detected 12 and 11 taxa, respectively (Fig. 4a), this approach was excluded from further analysis.

### Statistical analysis

Statistical analyses and data visualization were conducted in R (v4.0.4) [92] with packages such as Nonpareil, vegan, ape, ggplot2 [93], and dplyr. Principal coordinate analysis (PCoA) based on Jaccard metric was performed to demonstrate beta diversity [58]. Differences between groups were determined by Student’s *T*-test or ANOVA coupled with Tukey’s post hoc test, depending on the number of groups under comparison. *p* ≤ 0.05 was defined as statistically significant. Significance codes are as follows: *p* > 0.05 (ns), 0.01 < *p* ≤ 0.05 (*), 0.001 < *p* ≤ 0.01 (**), and *p* ≤ 0.001 (***). Package “fitdistrplus” was implemented to find the best-fit distribution for Nonpareil diversity. Machine learning models were trained using the package “caret.”

## Supplementary Information

**Additional file 1: Figure S1.** a) DNA yields of low-biomass surface-associated samples extracted by different methods. b) Practical relationship between reads number and submitted DNA input. **Figure S2.** Effects of whole-cell filtration on detected proportions of a) overall eukaryota reads, b) eukaryotes unassociated to humans, and c) human−associated reads. **Figure S3.** Whole-cell filtration did not have a significant impact on a) the overall number of recoverable taxa, b) the detectable number of locally abundant taxa or c) moderate taxa. **Figure S4.** Relative abundance of the top abundant taxa (average abundance > 1%) for samples with and without filtration. **Figure S5.** Biomass reduction with PMA treatment for samples and external standards, according to a) DNA quantity and b) 16S rRNA gene copy number. **Figure S6.** a) Cullen and Frey graph showing the distance from theoretical distributions to the observation. b) Fit for the normal distribution. c) Fit for the logistic distribution. **Figure S7.** Ranking of variables based on their explanatory power according to the R-squared value of a linear regression model. **Figure S8.** Variable importance rankings with and without the variable "study" based on random forest classifications. **Figure S9.** a) Distribution of the percentage of eukaryotic reads among 874 samples from hospital-related environmental studies. b) Surface samples, especially c) high-touch surface samples are more likely to contain higher proportions of eukaryotic reads. d) Distribution of the percentage of eukaryotic reads among 763 samples from high-touch surfaces. **Figure S10.** a) Experimental pipeline and b) result of the PMA validation experiment. **Figure S11.** Experimental pipeline for assessing and optimizing techniques in sample treatments. **Figure S12.** Schematic of the whole-cell filtration workflow.

**Additional file 2: Table S1.** Dataset of hospital-related environmental metagenomic samples used in the machine learning models.

**Additional file 3: Table S2.** Sample collection details.

**Additional file 4: Table S3.** Statistics of the genome-centric approach.

## Data Availability

The raw shotgun metagenomic sequencing data are available in the NCBI SRA repository under BioProject number PRJNA765404. Source code and supplementary data for reproducing analyses are available under MIT license at https://github.com/jxshen311/workflow_metagenomic_environmental_surveillance. Protocols are available at Protocol Exchange with DOIs: 10.21203/rs.3.pex-1656/v1 (sample collection, extraction, and cultivation), 10.21203/rs.3.pex-1657/v1 (snap freezing), 10.21203/rs.3.pex-1659/v1 (PMA treatment), and 10.21203/rs.3.pex-1658/v1 (DNA extraction).
