# Supplementary material for: An improved workflow for accurate and robust healthcare environmental surveillance using metagenomics
Source: Microbiome. 2022 Dec 2;10:206. doi: 10.1186/s40168-022-01412-x (PMC9716758; doi:10.1186/s40168-022-01412-x)
Supplement: Supplementary file 2 — Additional file 1: Figure S1. a) DNA yields of low-biomass surface-associated samples extracted by different methods. b) Practical relationship between reads number and submitted DNA input. Figure S2. Effects of whole-cell filtration on detected proportions of a) overall eukaryota reads, b) eukaryotes unassociated to humans, and c) human−associated reads. Figure S3. Whole-cell filtration did not have a significant impact on a) the overall number of recoverable taxa, b) the detectable number of locally abundant taxa or c) moderate taxa. Figure S4. Relative abundance of the top abundant taxa (average abundance > 1%) for samples with and without filtration. Figure S5. Biomass reduction with PMA treatment for samples and external standards, according to a) DNA quantity and b) 16S rRNA gene copy number. Figure S6. a) Cullen and Frey graph showing the distance from theoretical distributions to the observation. b) Fit for the normal distribution. c) Fit for the logistic distribution. Figure S7. Ranking of variables based on their explanatory power according to the R-squared value of a linear regression model. Figure S8. Variable importance rankings with and without the variable "study" based on random forest classifications. Figure S9. a) Distribution of the percentage of eukaryotic reads among 874 samples from hospital-related environmental studies. b) Surface samples, especially c) high-touch surface samples are more likely to contain higher proportions of eukaryotic reads. d) Distribution of the percentage of eukaryotic reads among 763 samples from high-touch surfaces. Figure S10. a) Experimental pipeline and b) result of the PMA validation experiment. Figure S11. Experimental pipeline for assessing and optimizing techniques in sample treatments. Figure S12. Schematic of the whole-cell filtration workflow. [file 40168_2022_1412_MOESM1_ESM.pdf]

Supplementary figures S1-S12

An improved workflow for accurate and robust healthcare environmental surveillance using metagenomics

Jiaxian Shen[1,*], Alexander G. McFarland[1], Ryan A. Blaustein[2], Laura J. Rose[3], K. Allison Perry-Dow[3], Anahid A. Moghadam[1], Mary K. Hayden[4], Vincent B. Young[5], Erica M. Hartmann[1]

[1]Department of Civil and Environmental Engineering, Northwestern University, USA

[2]Department of Nutrition and Food Science, University of Maryland, USA

[3]Centers for Disease Control and Prevention, USA

[4]Department of Internal Medicine, Division of Infectious Diseases, Rush Medical College, USA

[5]Department of Internal Medicine/Division of Infectious Diseases, The University of Michigan Medical School, USA

*Correspondence: Jiaxian Shen; 2145 Sheridan Road, Tech A223, Evanston, IL 60208-3109; Email: jiaxianshen2022@u.northwestern.edu; Tel: 872-985-1717

Supplementary information includes 12 figures and 3 tables.

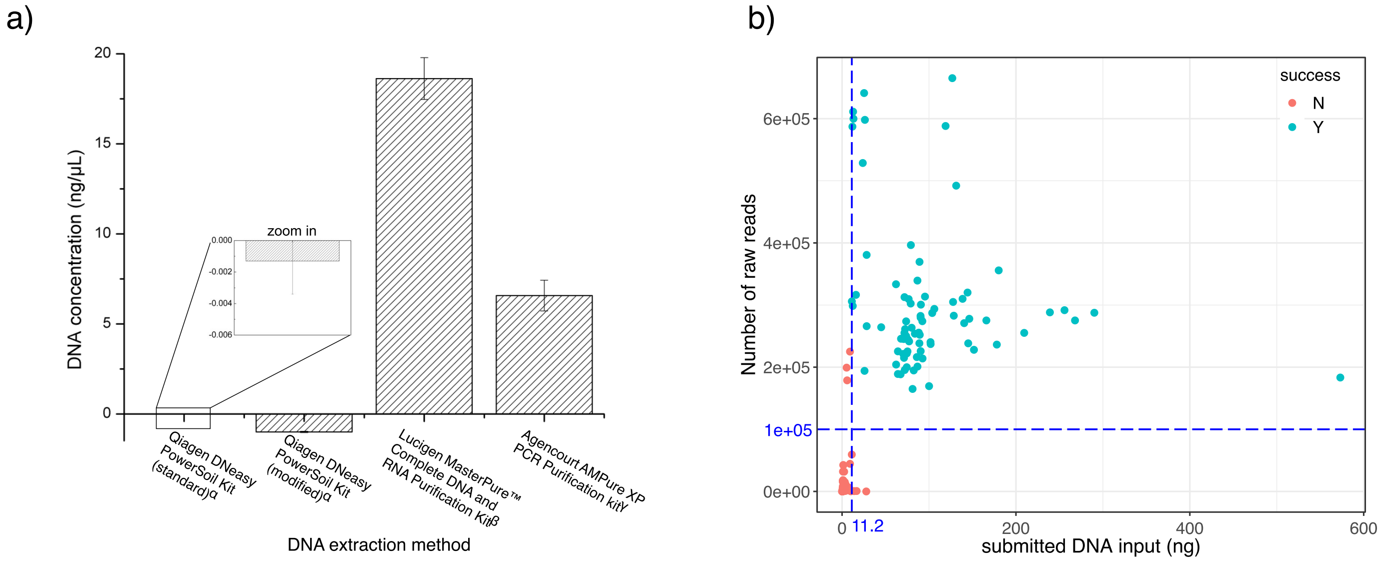

Fig. S1: a) DNA yields (quantified by NanoDrop) of low-biomass surface-associated samples extracted by different methods. Extraction kits marked with α are column-based, with β are magnetic bead-based, and with γ use liquid-liquid extraction. b) Practical relationship between reads number and submitted DNA input.

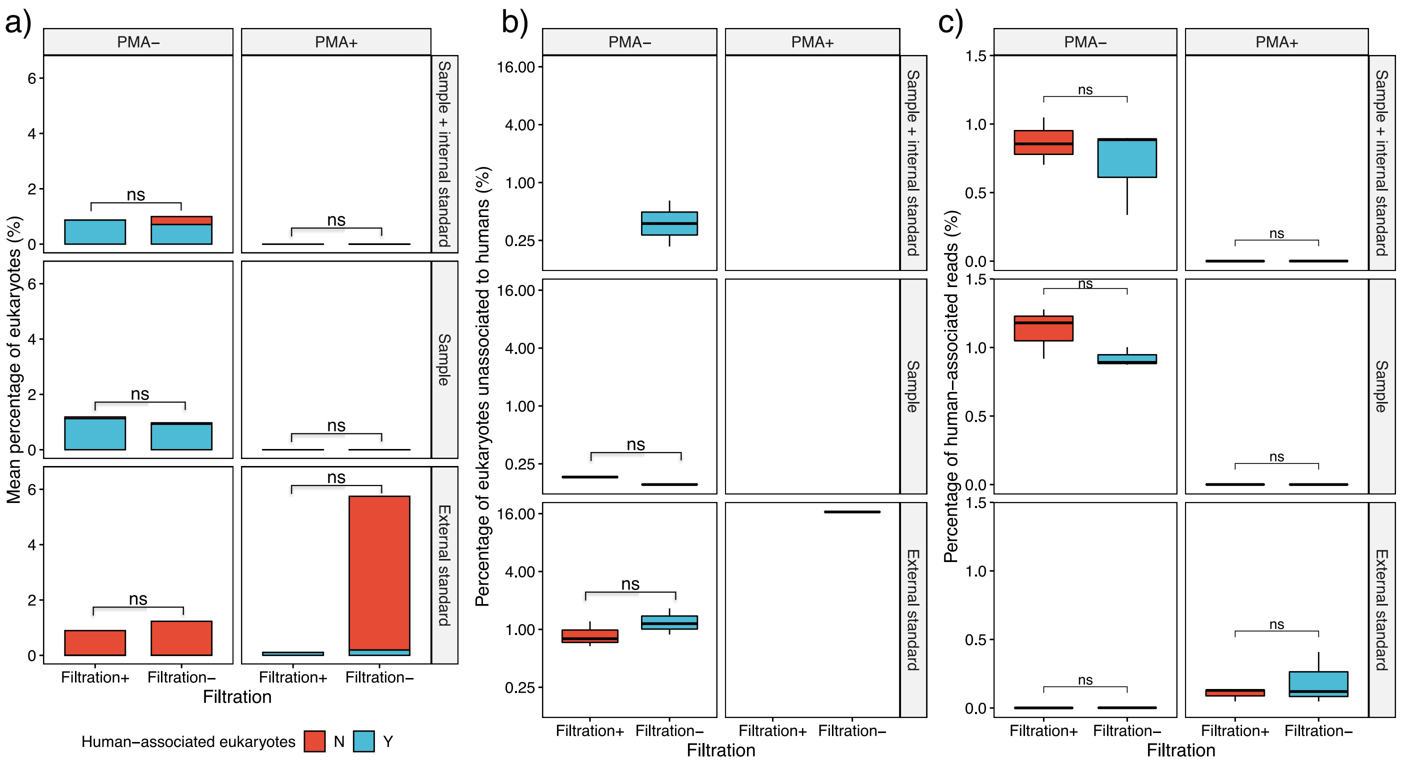

Fig. S2: Effects of whole-cell filtration on detected proportions of a) overall eukaryota reads, b) eukaryotes unassociated to humans, and c) human−associated reads. Significance was determined by paired t tests with BH corrections.

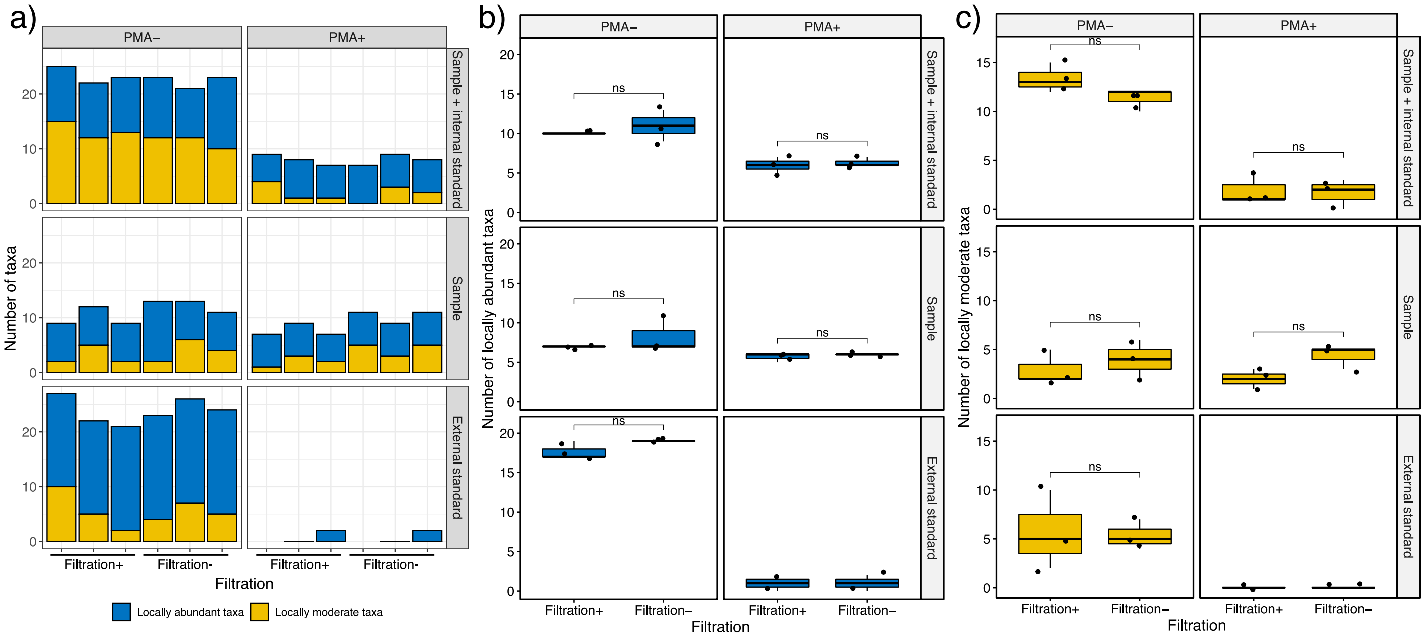

Fig. S3: Whole-cell filtration did not have a significant impact on a) the overall number of recoverable taxa, b) the detectable number of locally abundant taxa or c) moderate taxa. Significance was determined by paired t tests with BH corrections. Locally abundant, rare, and moderate taxa were defined as taxa with a representation of ≥1%, <0.01%, and < 1% & ≥ 0.01% within a sample.

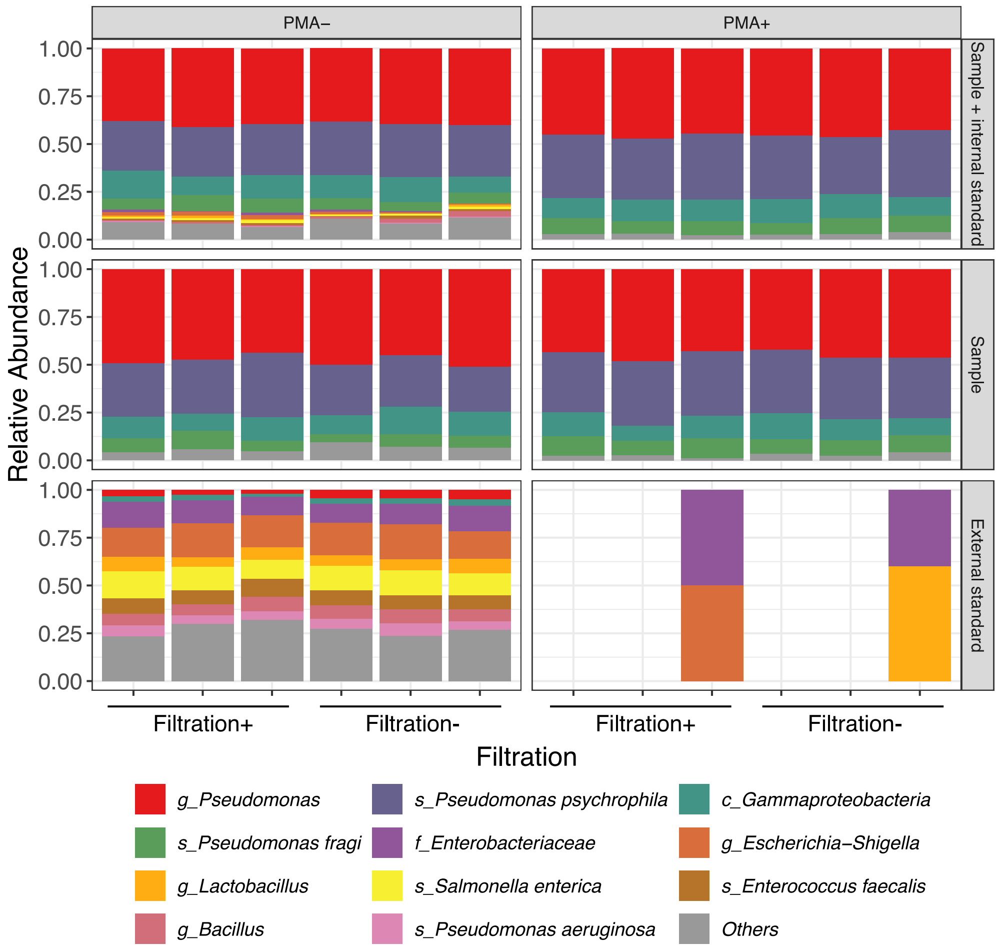

Fig. S4: Relative abundance of the top abundant taxa (average abundance > 1%) for samples with and without filtration.

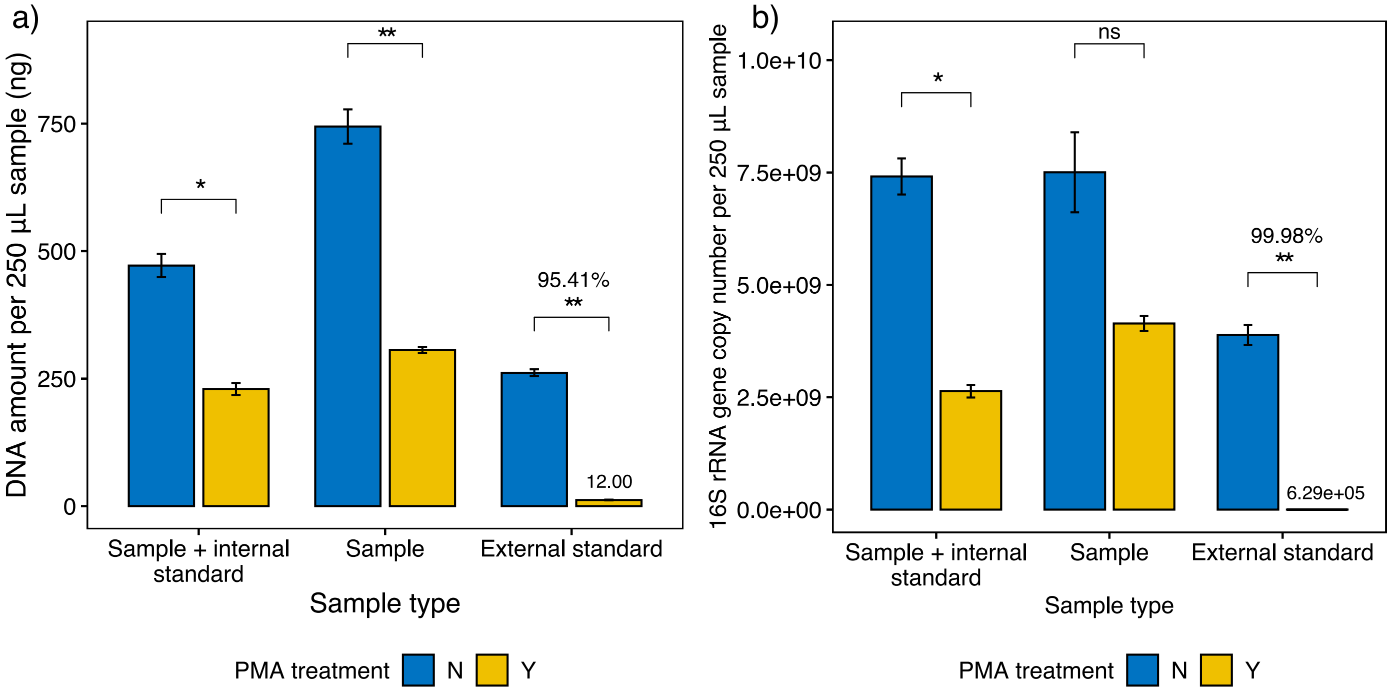

Fig. S5: Biomass reduction with PMA treatment for samples (with and without internal standards spiked in) and external standards, according to a) DNA quantity and b) 16S rRNA gene copy number. Significance was determined by paired t tests with BH corrections.

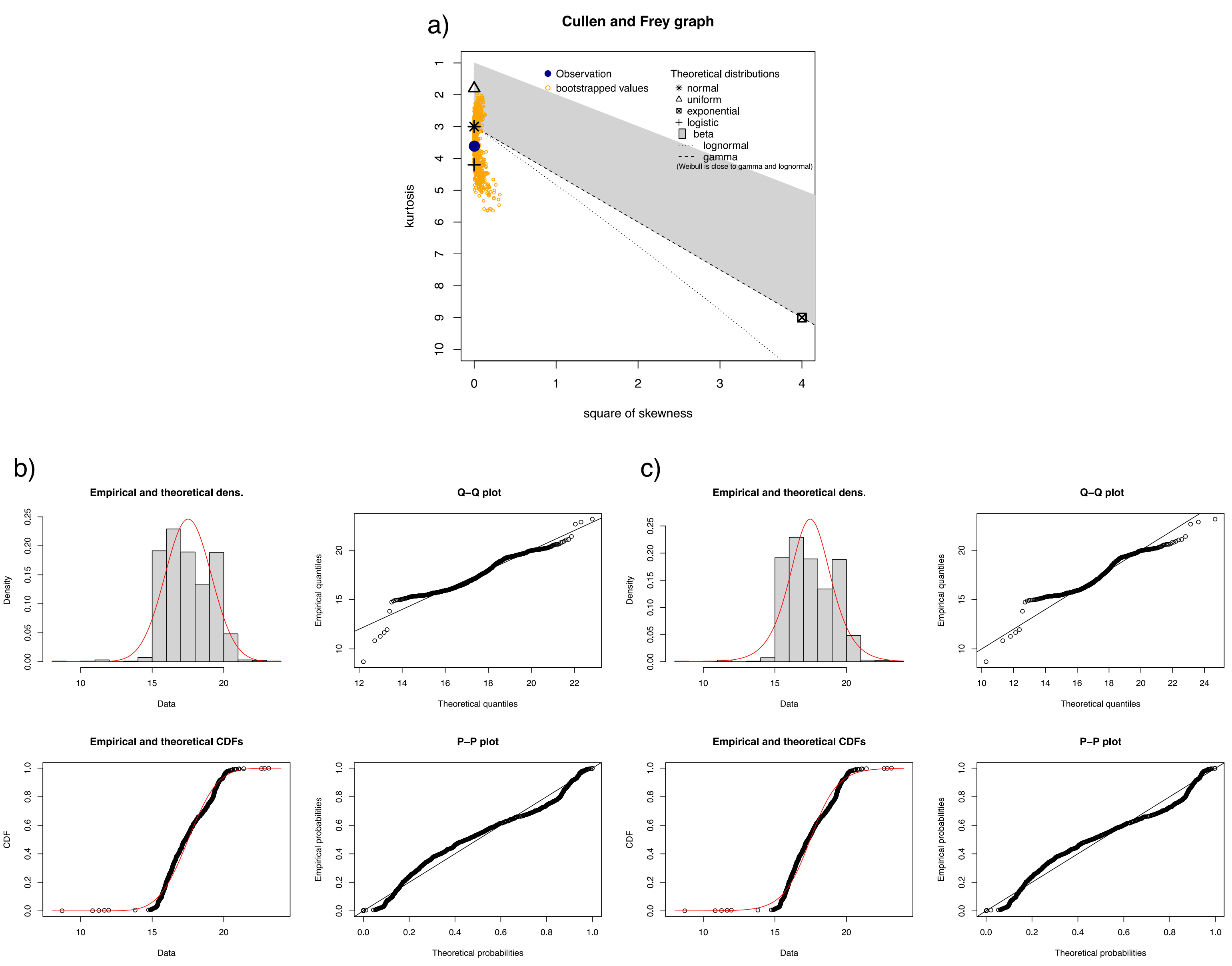

Fig. S6: a) Cullen and Frey graph showing the distance from theoretical distributions to the observation. b) Fit for the normal distribution. c) Fit for the logistic distribution.

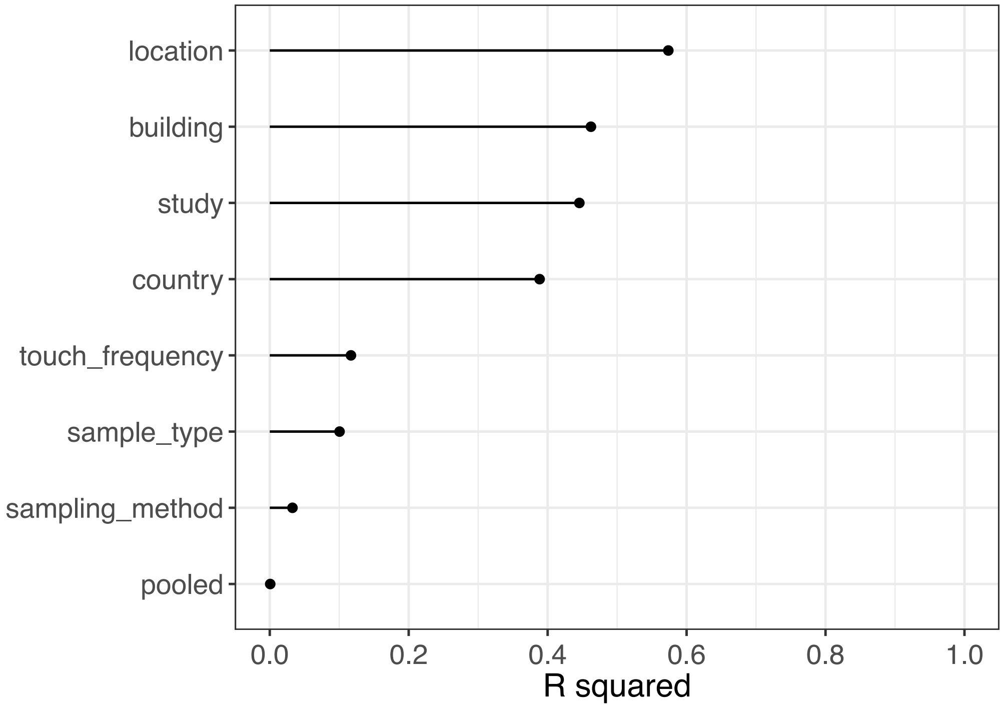

Fig. S7: Ranking of variables based on their explanatory power according to the R-squared value of a linear regression model.

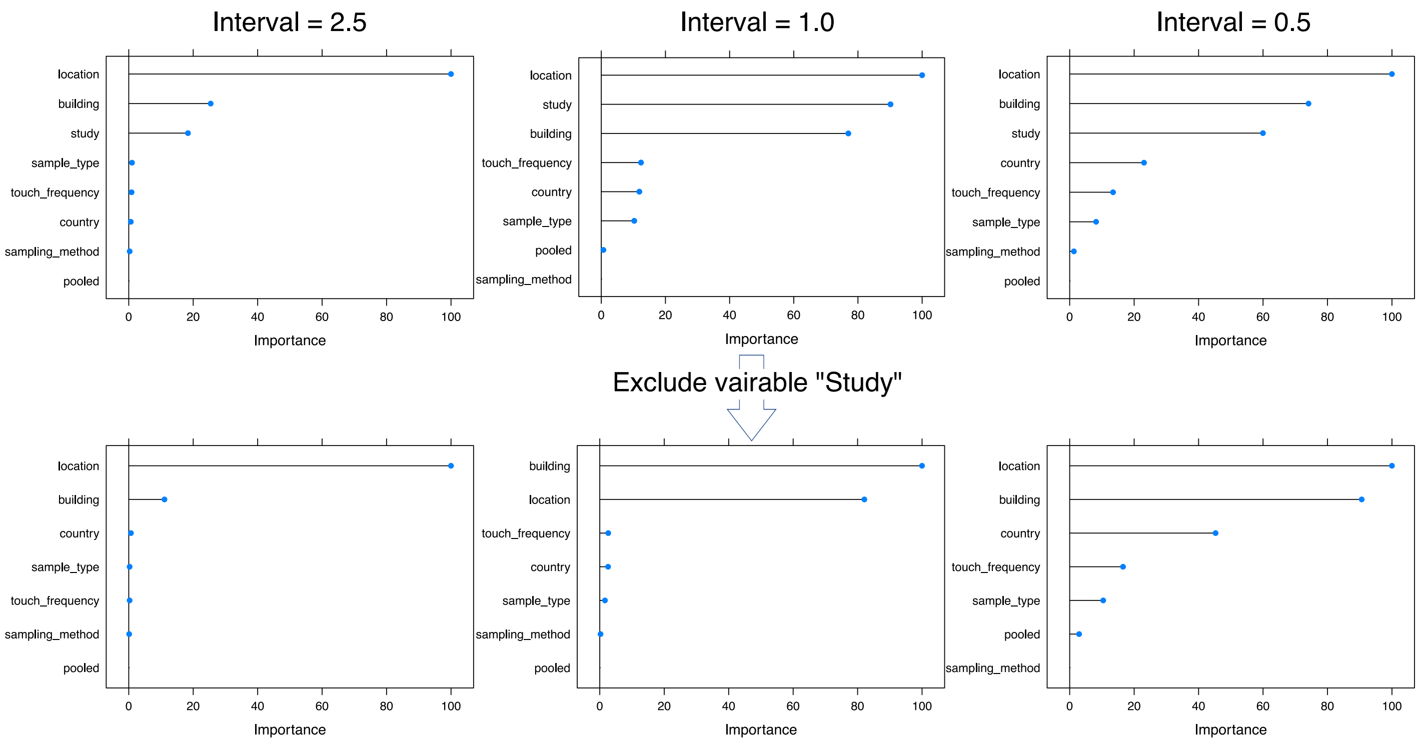

Fig. S8: Variable importance rankings with and without the variable "study" based on random forest classifications.

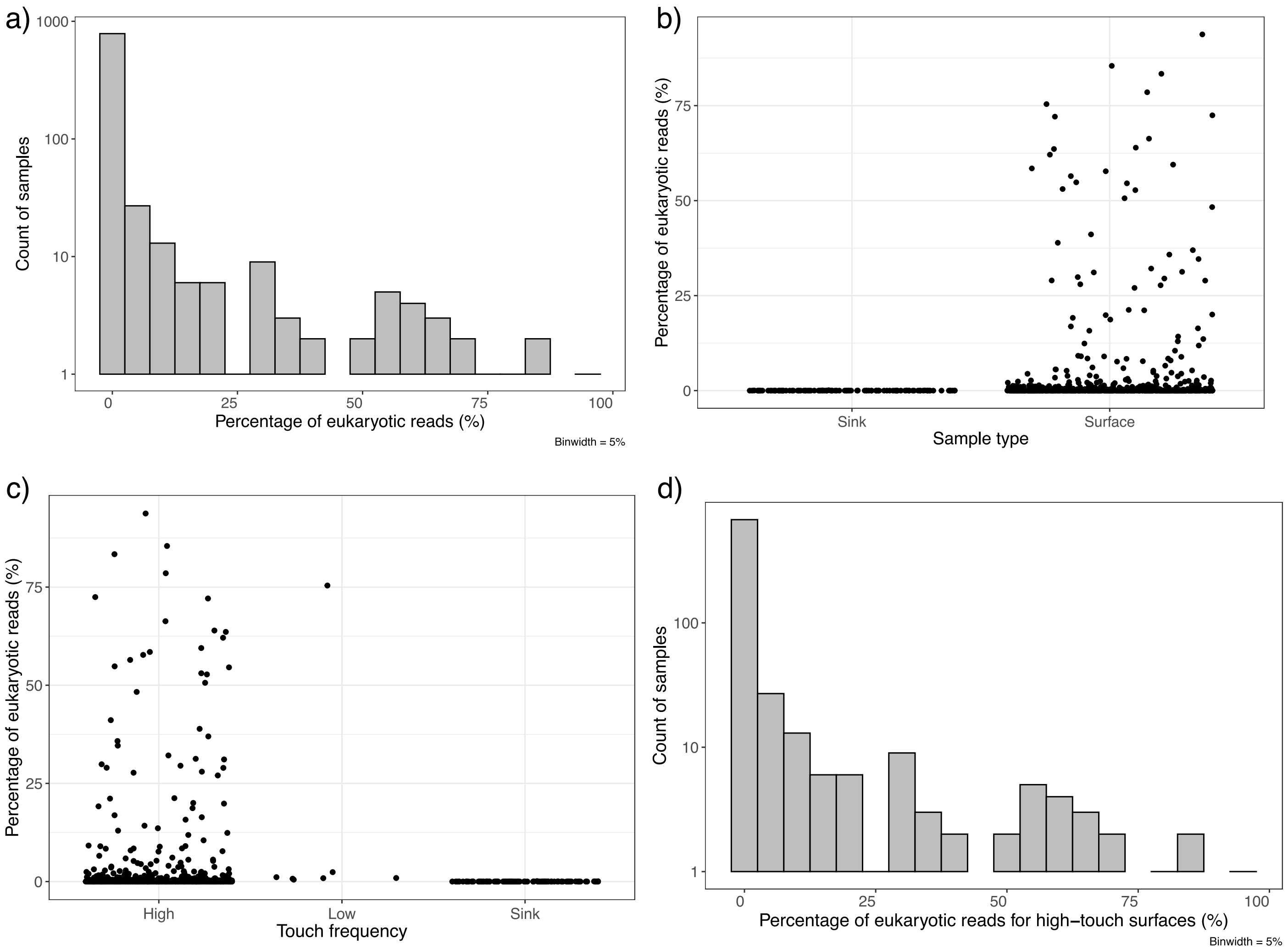

Fig. S9: a) Distribution of the percentage of eukaryotic reads among 874 samples from hospital-related environmental studies. b) Surface samples, especially c) high-touch surface samples are more likely to contain higher proportions of eukaryotic reads. d) Distribution of the percentage of eukaryotic reads among 763 samples from high-touch surfaces. Note that the percentage of eukaryota reads may be underestimated because we did not know whether these fastq files accessed from databases had been processed with tools like kneaddata or not.

a)

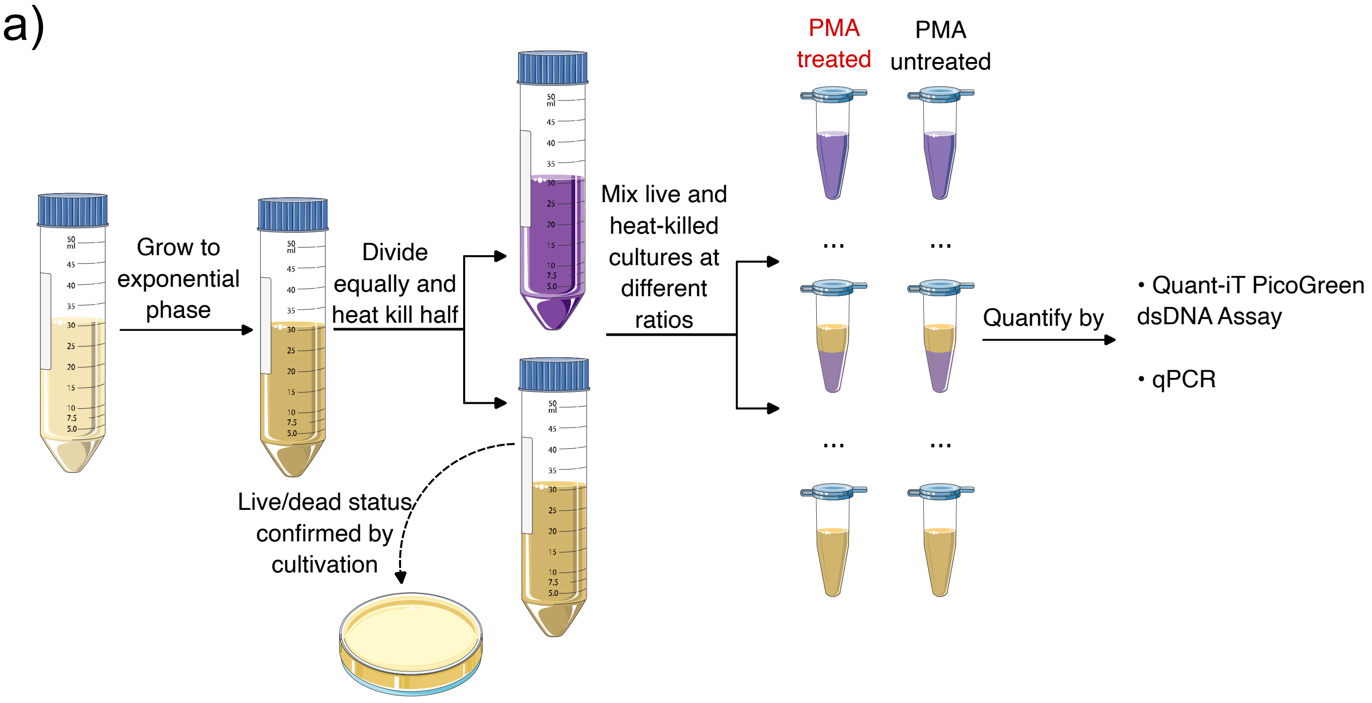

b)

$$\frac{T}{U} = \frac{\text{PMA } treated}{\text{PMA untreated}}$$

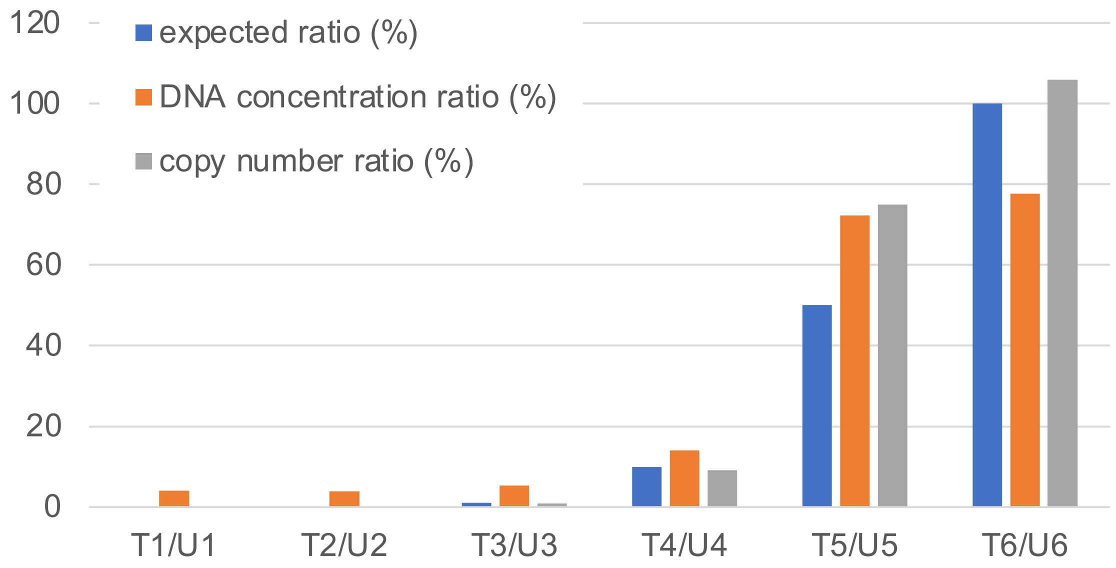

**Expected ratio (%)**	0	0.1	1	10	50	100
**DNA concentration ratio (%)**	4.11	3.94	5.39	14.12	72.27	77.72
**Copy number ratio (%)**	0.07	0.14	0.92	9.08	75.01	105.98

* Copy number ratio is calculated by assuming the efficiency equals 100%.

Fig. S10: a) Experimental pipeline and b) result of the PMA validation experiment. We want to acknowledge Servier (https://smart.servier.com/) for providing the following icons (licensed under CC-BY 3.0): microtube-closed-blue, falcon-50ml-pink, and petri-dish-yellow. Colors of the first two icons were changed to adapt to our needs.

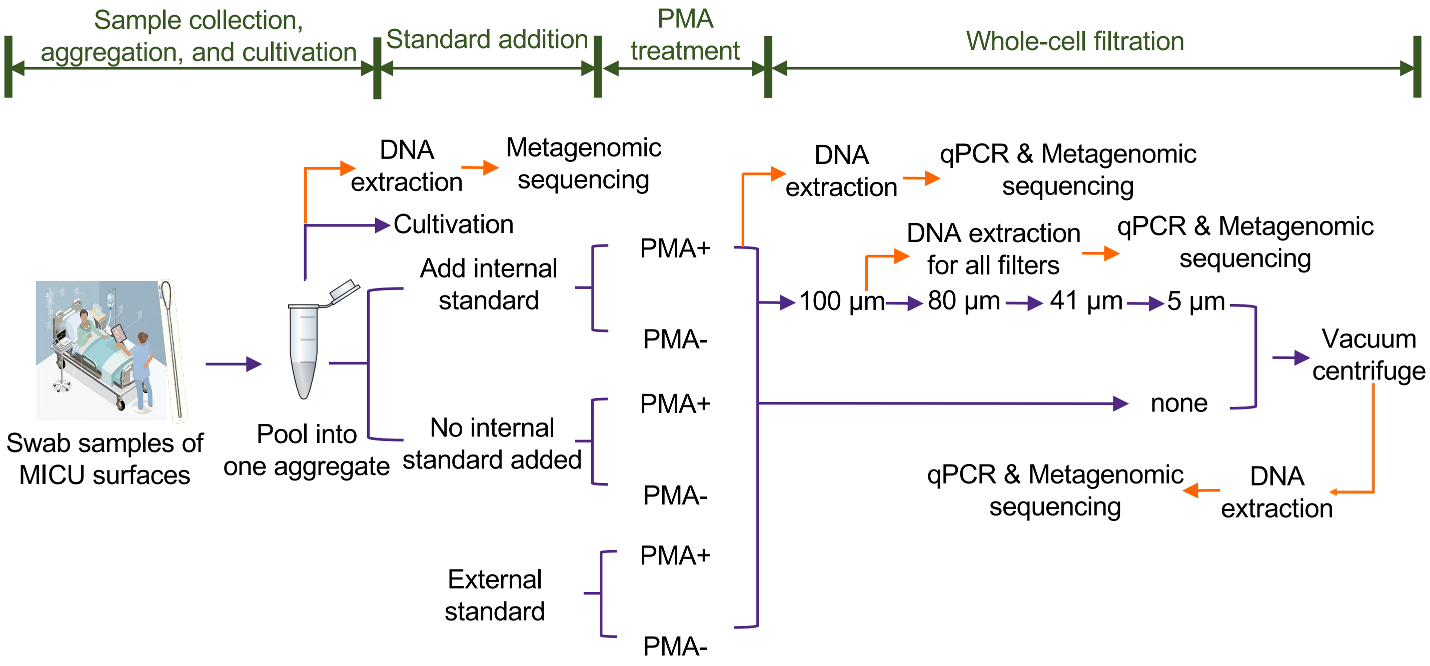

Fig. S11: Experimental pipeline for assessing and optimizing techniques in sample treatments.

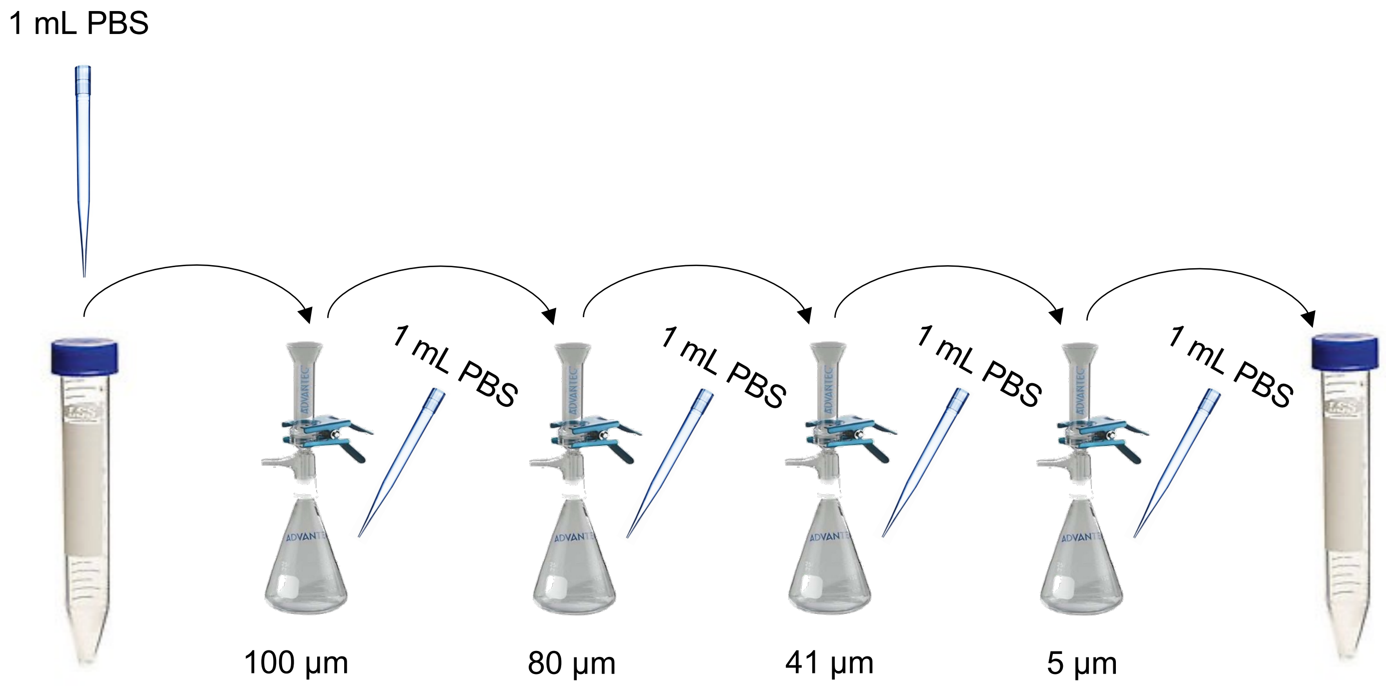

Fig. S12: Schematic of the whole-cell filtration workflow.
